# Paricalcitol Has a Potent Anti-Inflammatory Effect in Rat Endothelial Denudation-Induced Intimal Hyperplasia

**DOI:** 10.3390/ijms25094814

**Published:** 2024-04-28

**Authors:** Ciro Baeza, Arancha Pintor-Chocano, Susana Carrasco, Ana Sanz, Alberto Ortiz, Maria Dolores Sanchez-Niño

**Affiliations:** 1 Department of Vascular Surgery, IIS-Fundacion Jimenez Diaz UAM, 28040 Madrid, Spain; cbaezab@fjd.es; 2 RICORS2040, 28049 Madrid, Spain; 3 Department of Nephrology and Hypertension, IIS-Fundacion Jimenez Diaz UAM, 28040 Madrid, Spain; 4 Departamento de Medicina, Facultad de Medicina, Universidad Autónoma de Madrid, 28049 Madrid, Spain; 5 Departamento de Farmacología, Facultad de Medicina, Universidad Autónoma de Madrid, 28049 Madrid, Spain

**Keywords:** peripheral vascular disease, intimal hyperplasia, vitamin D receptor, paricalcitol, inflammation

## Abstract

Neointimal hyperplasia is the main cause of vascular graft failure in the medium term. Vitamin D receptor activation modulates the biology of vascular smooth muscle cells and has been reported to protect from neointimal hyperplasia following endothelial injury. However, the molecular mechanisms are poorly understood. We have now explored the impact of the selective vitamin D receptor activator, paricalcitol, on neointimal hyperplasia, following guidewire-induced endothelial cell injury in rats, and we have assessed the impact of paricalcitol or vehicle on the expression of key cell stress factors. Guidewire-induced endothelial cell injury caused neointimal hyperplasia and luminal stenosis and upregulated the expression of the growth factor growth/differentiation factor-15 (GDF-15), the cytokine receptor CD74, NFκB-inducing kinase (NIK, an upstream regulator of the proinflammatory transcription factor NFκB) and the chemokine monocyte chemoattractant protein-1 (MCP-1/CCL2). Immunohistochemistry confirmed the increased expression of the cellular proteins CD74 and NIK. Paricalcitol (administered in doses of 750 ng/kg of body weight, every other day) had a non-significant impact on neointimal hyperplasia and luminal stenosis. However, it significantly decreased GDF-15, CD74, NIK and MCP-1/CCL2 mRNA expression, which in paricalcitol-injured arteries remained within the levels found in control vehicle sham arteries. In conclusion, paricalcitol had a dramatic effect, suppressing the stress response to guidewire-induced endothelial cell injury, despite a limited impact on neointimal hyperplasia and luminal stenosis. This observation identifies novel molecular targets of paricalcitol in the vascular system, whose differential expression cannot be justified as a consequence of improved tissue injury.

## 1. Introduction

Intimal hyperplasia is the pathological process that underlies arterial restenosis after endothelial damage, and it develops as a result of the migration of vascular smooth muscle cells (VSMCs) [1,2]. It is the main cause of vascular graft failure in the medium term [3,4,5]. A correct understanding of its molecular mechanisms and modulators may improve the outcomes of endovascular procedures. Vitamin D is an important regulator of calcium metabolism, but also has pleiotropic effects. For example, it has a significant antiproliferative effect on some cellular systems [6]. This antiproliferative effect could decrease VSMC proliferation and intimal hyperplasia secondary to endothelial injury. Most studies on the antiproliferative effect of vitamin D have been performed in malignancy [7]. However, vitamin D also modulates VSMC proliferation and epidermal growth factor (EGF) and elastin gene expression [6,8,9], all of which may reduce the development of intimal hyperplasia in vivo following vascular injury [10,11].

Paricalcitol is a third-generation vitamin D analog used to treat secondary hyperparathyroidism in patients with chronic kidney disease (CKD) [12,13]. It is considered a selective activator of the vitamin D receptor (VDR) that has a better safety profile regarding hypercalcemia than vitamin D. VDR is expressed by multiple cell types, including VSMC and immune system cells [14]. Paricalcitol has pleiotropic actions, including a cardiovascular system impact through regulation of VSMC proliferation and differentiation, thrombosis and fibrinolysis, vascular contraction and relaxation and endothelial regeneration [15]. Additionally, paricalcitol has anti-inflammatory effects in different cell types [12,16,17,18,19].

Given that vitamin D had previously been reported to protect from neointimal hyperplasia [10,11,20,21], although not by all authors [22], and that the neointima is rich in VDR [23], we studied the impact of paricalcitol on neointimal hyperplasia and potential molecular targets in rats. The information obtained may identify novel therapeutic targets for human disease.

## 2. Results

### 2.1. Paricalcitol, Intimal Hyperplasia and Stenosis Secondary to Endothelial Injury

**Figure 1:**
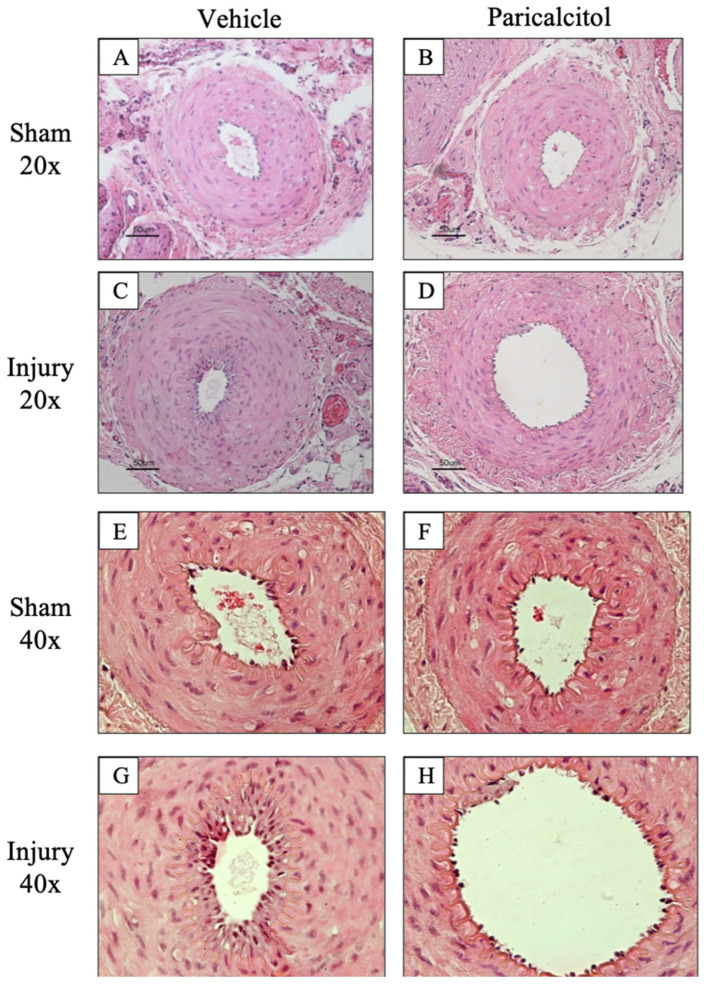
Increased intima–media ratio and stenosis following endothelial injury in rat femoral arteries. Endothelial injury was induced by scraping (injury artery) and a sham control without scraping was obtained from the contralateral artery. One group of rats received paricalcitol (750 ng/kg) and the other received the vehicle for 14 days, and artery samples were evaluated at 14 days. Images represent 20X (**A**–**D**) and 40X (**E**–**H**) magnification of hematoxylin eosin staining representative samples from the different groups. In panels **E**–**H**, intimal hyperplasia delimited by the internal elastic lamina can be better appreciated. Table 1 presents quantitative results for intima–media ratio and % stenosis.

Two weeks after injury, hematoxylin–eosin staining disclosed a normal appearance of the arteries that underwent a sham procedure, independently of treatment with vehicle or paricalcitol. Following endothelial denudation through guidewire injury, neointimal hyperplasia and luminal stenosis were noted (Table 1, Figure 1). There were no statistically significant differences between the vehicle and paricalcitol arteries. However, trends in stenosis were observed that were generally aligned with data published in the literature [10,20].

### 2.2. Endothelial Denudation-Induced Intimal Hyperplasia Is Associated with Increased Local Gene Expressions of GDF15, CD74, NIK and MCP-1

**Figure 2:**
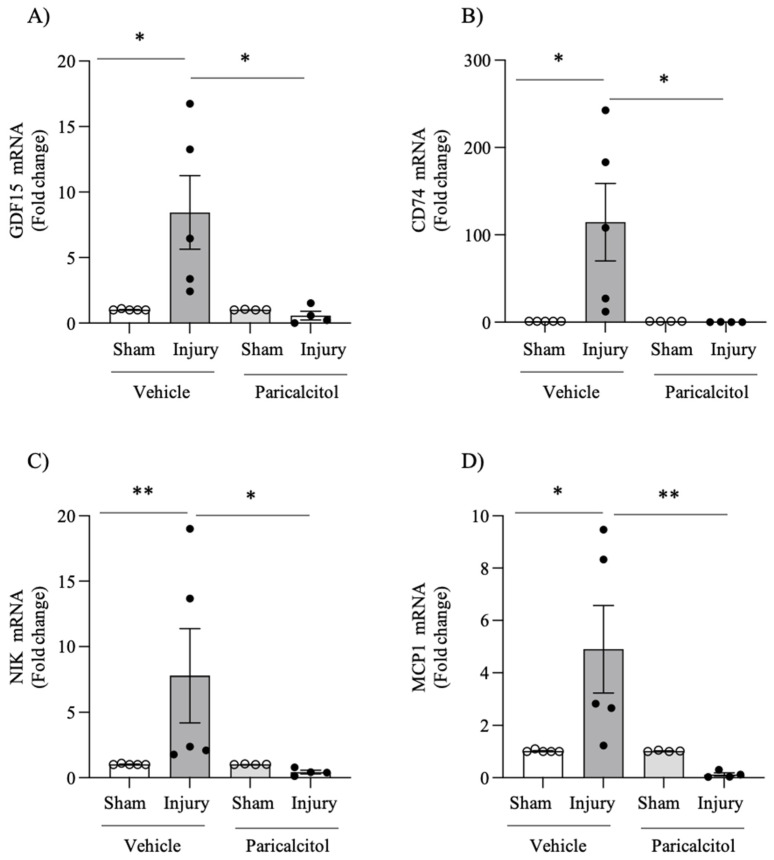
Increased femoral artery gene expression for key molecules: impact of paricalcitol. Arterial injury was associated with an increased mRNA expression of growth factors and inflammatory mediators, and this was prevented by paricalcitol. mRNA expression was studied using RT-qPCR. Data expressed as % increase over the sham artery, which was assigned a value of 100%. (**A**) GDF15. (**B**) CD74. (**C**) NIK. (**D**) MCP-1. * *p* < 0.01 vs. injured vehicle; ** *p* < 0.05 vs. injured vehicle.

Next, we explored potential mediators of tissue injury in intimal hyperplasia secondary to endothelial damage and the effect of paricalcitol. For this, we studied, in the most distal third of the arterial sample from each specimen, the gene expression at the mRNA level, using quantitative RT-PCR, of mediators of tissue damage with theoretical potential both to modulate tissue damage and to be therapeutic targets. GDF15 is a growth factor with tissue-protective and anti-inflammatory properties [24]. CD74 is a membrane receptor for the cytokines MIF (macrophage migration inhibitory factor) and DDT (D-dopachrome tautomerase, MIF-2) that are involved in atherogenesis and arterial remodeling secondary to endothelial injury [25,26]. NIK is the apical kinase of the non-canonical pathway for the prolonged activation of the proinflammatory transcription factor NFκB [27], while MCP1 is a well-characterized chemokine that attracts monocytes/macrophages [28]. Intimal hyperplasia secondary to endothelial damage was associated with increased mRNA expressions of GDF15, CD74, NIK and MCP-1, compared to the contralateral sham artery (Figure 2).

**Figure 3:**
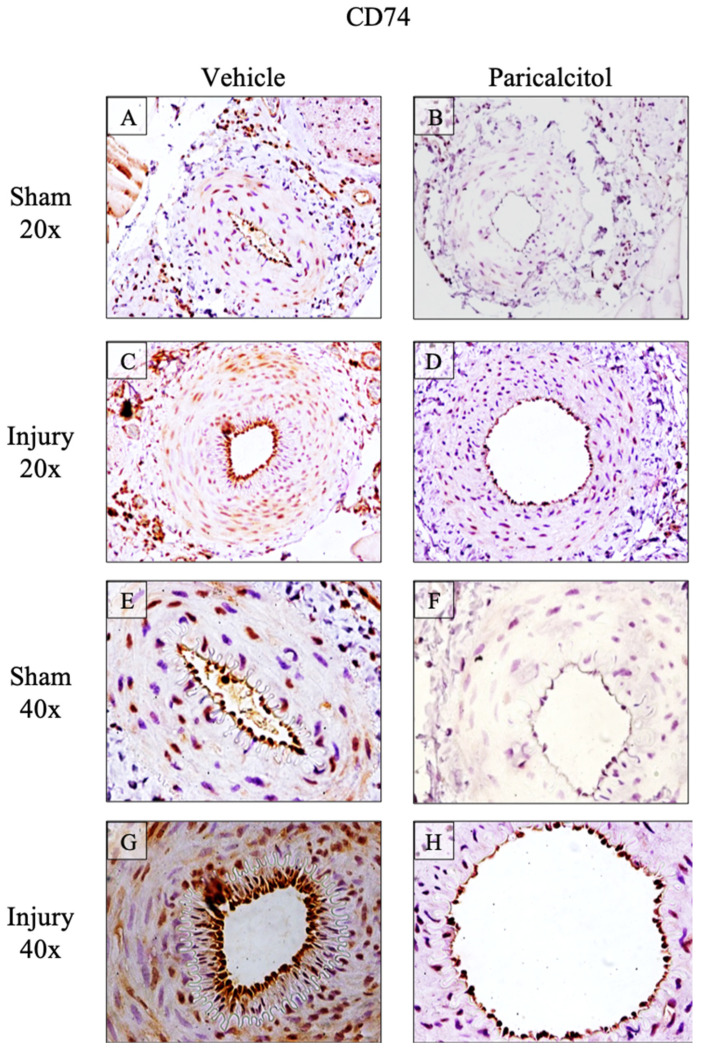
Increased femoral artery CD74 protein expression: impact of paricalcitol. Expression of CD74 in rat femoral arteries. Constitutive CD74 expression is observed in vehicle sham arteries (**A**,**E**) and is lower in paricalcitol sham arteries (**B**,**F**). CD74 expression increased in injured arteries, both in the media (**C**) and endothelium (**G**). Treatment with paricalcitol in injured arteries reduced CD74 expression to basal levels (**D**,**H**).

**Figure 4:**
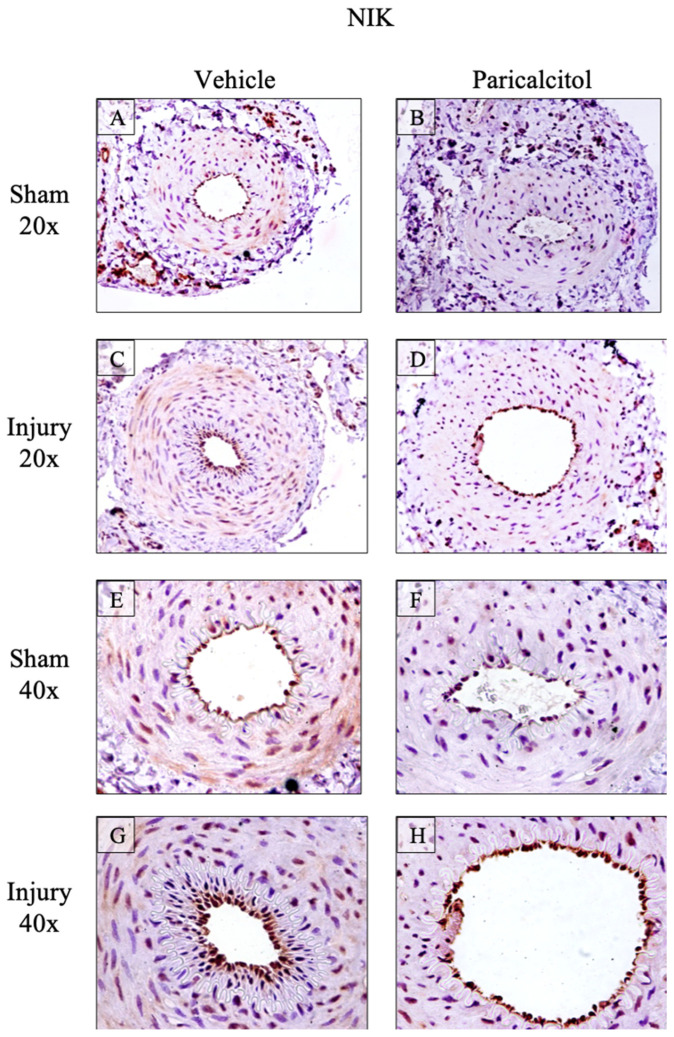
Increased femoral artery NIK protein expression: impact of paricalcitol. NIK expression in rat femoral arteries. Constitutive NIK expression is observed in vehicle sham arteries (**A**,**E**). In sham arteries of rats that received paricalcitol (**B**,**F**), NIK expression is lower in all layers of the artery. Injured arteries developing intimal hyperplasia (**C**,**G**) express NIK in endothelium and media. Paricalcitol decreased NIK expression in injured arteries (**D**,**H**).

The changes in gene expression were confirmed to result in changes in protein levels, using immunohistochemistry, for CD74 and NIK, which are intracellular proteins (i.e., they are not released into the extracellular space where they may become diluted or carried away by the circulation), although CD74 may also be expressed in the cell surface. CD74 was constitutively expressed throughout the arterial wall and the expression was increased in injured vehicle arteries and localized in the endothelium and media (Figure 3). NIK was also constitutively expressed, showing diffuse staining of the media (Figure 4). NIK was increased both in the endothelium and media of injured arteries.

### 2.3. Paricalcitol Blunts the Changes in Gene Expression of Growth Factors and Mediators of Inflammation

Treatment with paricalcitol decreased the expression of GDF-15, CD74, NIK and MCP-1 mRNA in injured arteries to levels found in sham arteries (Figure 2). At the protein level, paricalcitol decreased the expression of CD74 in the media and limited it to the endothelial layer (Figure 3). Paricalcitol decreased NIK expression both in sham healthy arteries, where expression of the NIK protein was practically not observed, and in injured arteries, in which NIK expression decreased in the endothelium and in the media (Figure 4).

### 2.4. Paricalcitol Prevents Vascular Inflammation during Endothelial Denudation-Induced Intimal Hyperplasia

**Figure 5:**
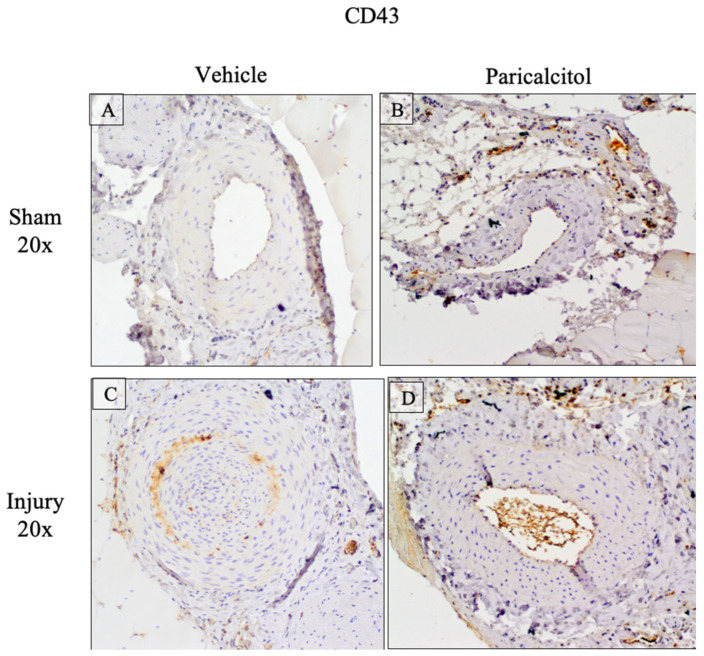
Increased femoral artery infiltration by CD43-expressing leukocytes: impact of paricalcitol. CD43 protein expressing leukocytes in rat femoral arteries (×20), either sham (**A**,**B**) or injured (**C**,**D**). CD43 staining showed leukocyte infiltrates in vehicle injured arteries (**C**). This infiltrate was lower in paricalcitol-treated injured (**D**) arteries.

In injured arteries of control rats, the number of CD43-expressing leukocytes increased (Figure 5). Treatment with paricalcitol decreased the leukocyte infiltrate (Figure 5), which is consistent with the reduced expression of NIK, an upstream regulator of NFĸB, and the chemokine MCP-1.

## 3. Discussion

The main finding is that endothelial cell injury causing intimal hyperplasia results in the recruitment of an arterial wall stress response, characterized by an increased expression of growth factors, such as GDF15, cytokine receptors, such as CD74, upstream regulators of the proinflammatory transcription factor NFĸB, such as the NIK kinase, and of chemokines, such as MCP1. This was associated with local inflammation. Paricalcitol decreased this stress and proinflammatory response, despite a milder impact on intimal hyperplasia than previously reported in the literature of other VDR activators. The mild impact on histomorphometric tissue injury supports the concept that these genes may be direct paricalcitol targets and the changes in gene expression are not secondary to an overall improvement in tissue injury, a concept that should be validated by molecular biology investigations.

Vitamin D had previously been reported to protect from neointimal hyperplasia [10,11,20,21], although not by all authors [22]. However, the therapeutic use of vitamin D is marred by potential adverse effects on calcium–phosphate metabolism, as overdosing can cause hypercalcemia, hyperphosphatemia, hypercalciuria, urolithiasis, nephrocalcinosis and kidney failure [29,30,31,32]. Thus, VDR agonists, such as paricalcitol, that have a milder impact on serum calcium and phosphate [33], may be of interest to design novel clinical studies. However, PubMed searches using the key words paricalcitol and hyperplasia or endothelial did not find reports on the impact of paricalcitol on endothelial cell injury causing intimal hyperplasia. More recently, paricalcitol was reported to protect from endothelial cell injury and attenuate endothelial dysfunction [34,35,36] and treatment with vitamin D improves endothelial dysfunction in patients with kidney disease [37,38]. We have now addressed the vascular protective effect of paricalcitol in preclinical endothelial denudation-induced intimal hyperplasia. The observed impact on stenosis was generally aligned with results reported in the literature for most vitamin D studies: a trend towards benefit was observed that did not reach statistical significance. However, this relative disappointment is a key strength of the current dataset: despite the mild impact on overall vascular injury, there were dramatic changes in the expression of several genes involved in tissue injury, tissue protection and inflammation, suggesting that these changes in gene expression are a direct consequence of paricalcitol therapy and do not reflect a general improvement in tissue injury. In this regard, the VDR transcription factor is activated by ligand binding [39]. Overall, paricalcitol and active vitamin D3 (calcitriol) had similar effects on gene expression in human VSMC: 115 to 116 genes were up-regulated and 60 to 61 genes down-regulated [15]. It also modulates gene expression in other key cell types for vascular injury, such as inflammatory cells [40].

Neither GDF-15 nor CD74, NIK or MCP-1/CCL2 are known targets of the VDR [41] (accessed on 25 December 2023), so the present information is novel. The fact that expression of these genes is suppressed by paricalcitol under stress conditions may explain why there are no considered canonical VDR targets. Alternatively, gene expression in response to VDR activation may be regulated through at least 47 transcription factors that are primary VDR targets [40] or through non-canonical, VDR-independent signaling [42]. However, data derived from other cell systems already hinted to GDF15, CD74 and MCP1 being VDR and/or paricalcitol targets. Thus, paricalcitol had been previously shown to suppress CD74 and MCP1 expression in stressed podocytes, as a part of a wider anti-inflammatory response [16,43]. Paricalcitol also prevented the downregulation of TNF receptor-associated factor 3 (TRAF3) in human peripheral blood mononuclear cells, resulting in decreased noncanonical NFκB-mediated proinflammatory responses, an effect that was shown to be VDR-independent in VDR knockout mice [12]. Additionally, calcitriol decreased GDF15 expression in human colon adenocarcinoma samples, although the effect of paricalcitol was not studied [44]. By contrast, calcitriol increased GDF15 expression in human prostate cancer cells [45]. Finally, we did not find evidence that an impact of the VDR or paricalcitol on NIK expression had been previously studied. NIK is encoded by the MAP3K14 gene and is an upstream regulator of the noncanonical activation of the proinflammatory transcription factor NFκB [27]. Non-canonical activation confers durability to the otherwise transient activation of NFκB. NIK overactivity promotes tissue injury in multiple tissues and organs and appears to contribute to vascular calcification and endothelial dysfunction [27,46]. Decreased NIK expression may represent a novel molecular mechanism for the vascular protective effect of paricalcitol.

Both GDF15 and CD74 may have both context-dependent tissue-protective and tissue-damaging effects, and it is unclear whether their suppression by paricalcitol may have contributed to milder vascular injury following endothelial denudation or could have contributed to the absence of significant impact on intimal hyperplasia. While GDF15 has tissue-protective properties, as shown in acute kidney injury and chronic kidney disease [24], it promoted vascular injury and GDF15 deficiency protected against brachiocephalic trunk lumen stenosis after a cholesterol-enriched diet in ApoE−/− mice [47,48]. CD74 was initially thought to be involved in tissue injury, as a receptor for chemokines known to cause tissue injury whose expression is increased in stressed tissues [49]. In vessels, CD74 is expressed at increased levels in plaques and peripheral blood mononuclear cells from patients with carotid stenosis and was associated with intima–media thickness in subjects free from clinical cardiovascular diseases [26]. Among multiple reports supporting the involvement of CD74 or its receptors in tissue injury, CD74 ablation protects against T2D-induced cardiac remodeling and contractile dysfunction [50]. However, CD74 has additional functions as an intracellular protein and has co-receptors that may modify its function, yielding tissue-protective properties [49,51]. As an example, CD74 is part of a pro-survival pathway, in epithelial cells triggered in a compensatory manner in response to inflammation, and also has other protective functions [51,52,53].

Some limitations should be acknowledged, including the fact that preclinical data were not confirmed in human tissue samples. However, this would have required a randomized clinical trial with invasive procedures. Furthermore, while the impact of paricalcitol, a therapeutic agent in clinical use, was studied, whether paricalcitol effects were mediated only through activation of the VDR, which would have required studies in VDR deficient animals, was not explored. Finally, the specific function in endothelial denudation-induced vascular injury of the molecules studied was not addressed in NIK-, CD74- or GDF15-deficient animals. Although we did not specifically assess VDR expression in rat tissues, VDR immunoreactivity is well known to be expressed in rat aorta endothelial and vascular smooth muscle cells [54]. Additionally, VDR is expressed in human endothelial and vascular smooth muscle cells [55].

In conclusion, paricalcitol suppressed the stress response to guidewire-induced endothelial cell injury, and resulted in suppressed features of inflammation, but had limited impact on neointimal hyperplasia and luminal stenosis. While disappointing from the point of view of the lack of a strong protective effect over histomorphometric parameters in vivo, this lack of a strong protective effect allowed the identification of novel molecular targets of paricalcitol in vascular injury, whose change in expression cannot be justified by a milder injury. By identifying paricalcitol targets which may have separate and even opposing effects on tissue injury, these results advance our understanding of the molecular mechanisms of paricalcitol-induced tissue protection and open the door to the future characterization of molecular targets and their individual impact on outcomes.

## 4. Materials and Methods

### 4.1. Study Protocol

**Figure 6:**
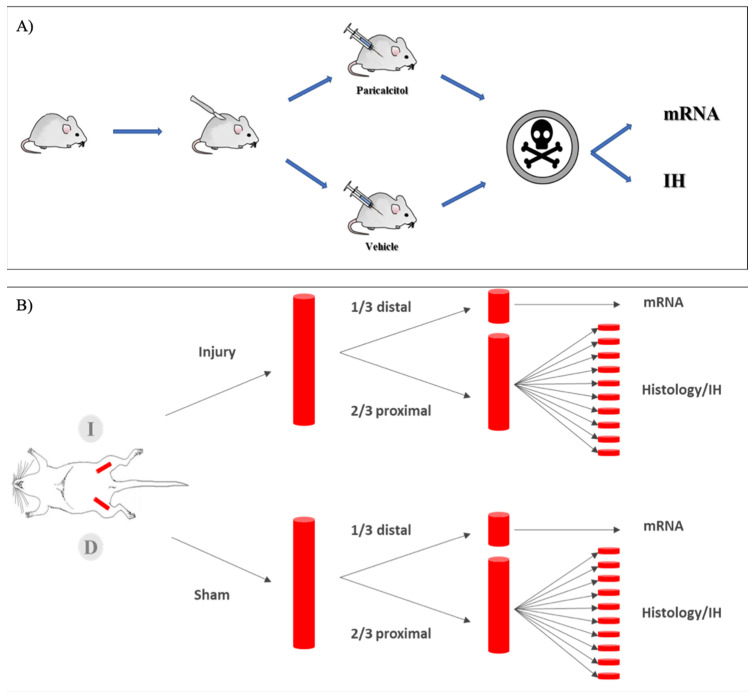
Experimental design. (**A**) Experimental model in rats. In each rat, the endothelium of the left femoral artery was injured by a vascular-guided scraping technique (injury artery). A similar procedure without scraping in the right femoral artery yielded sham artery controls. One group of rats received paricalcitol (750 ng/kg) and the other the vehicle for 2 weeks. Artery samples were evaluated 14 days after the intervention. Samples for mRNA, immunohistochemistry and histomorphometry were obtained from each arterial bundle. (**B**) Processing of arterial samples. After euthanasia, both femoral arteries were removed. The most distal third was used for molecular biology and protein studies. Histomorphometry and immunohistochemistry were performed in the proximal two thirds of the vascular bundle. To assess the degree of endothelial injury throughout the entire artery, this was divided into 10 consecutive sections that were stained and results averaged to yield the result of the sample.

Figure 6 summarizes the experimental approach. In summary, mechanical endothelial injury was performed in rat femoral arteries, and rats were treated with paricalcitol or vehicle. In artery samples, the endothelial lesion was studied using histomorphometry, mRNA expression was quantified using quantitative RT-PCR and cells expressing the protein products were located using immunohistochemistry.

### 4.2. Animal Model

Animal studies were performed in accordance with the Guidelines of the National Institutes of Health for the Care and Use of Laboratory Animals, respecting current national regulations. Two groups of five female 2-month-old Wistar Kyoto rats were studied, one group received paricalcitol (750 ng/kg of body weight every other day) intraperitoneally, starting immediately after the intervention for 2 weeks [16], and the other group received the vehicle (alcohol 20% *v*/*v*, propylene glycol and purified water). The dose of paricalcitol was based on prior experience that showed an anti-inflammatory effect in the kidney in rats [16]. Preclinical development of paricalcitol was initiated in rats, in which doses of 450 to 4500 ng/kg were shown to be safe and to suppress parathyroid hormone (PTH) [56]. The first human study tested doses of up to 160 ug, approximately over 2000 ng/kg [57]. However, currently, the initial dose of paricalcitol for secondary hyperparathyroidism is calculated, in mcg, based on baseline iPTH level in pg/mL divided by 80 [58]. Thus, the dose used in the present study in rats would be equivalent to the dose needed for someone with a baseline PTH level of over 4000 pg/mL, which is exceedingly high and usually not seen in the clinic.

Under inhaled isoflurane anesthesia, the femoral artery endothelium was denudated by scraping using a 0.014 “(0.35 mm) diameter angioplasty guide (1000462H: Abbott Hi-Torque Balance Heavyweight Guide Wire 0.014” × 190 cm). Under a surgical microscope, an incision was made on the inside of the leg at the level of the joint, over the femoral package. The femoral artery was carefully dissected for arteriotomy, controlled with 7/0 Silkam^®^ surgical floss, which can act as ligation. This was followed by a transverse scissor arteriotomy, distal to the silk. The guidewire was then inserted manually with the help of forceps, and advanced retrogradely to the level of the aortic bifurcation. Circular and antero-posterior rubbing movement was performed for 30 s before removing the guide. To prevent bleeding, the artery was ligated with proximally placed floss. A sham injury protocol was performed on the contralateral side, without passage of the guide. Endothelial injury was performed in the left femoral artery and the right femoral artery was used as sham surgery control. After euthanasia, perfusion was performed with 20 mL of 0.9% NaCl solution by direct injection into the left ventricle of the heart, facilitating its exit through an arteriotomy performed proximally to the femoral ligation. The femoral bundles were then removed up to the aortic bifurcation. For immunohistochemical processing, samples were immersed in 4% paraformaldehyde in Phosphate-buffered saline (PBS) buffer for subsequent inclusion in paraffin. The most distal part of the femoral arteries was immediately frozen in liquid nitrogen for protein and molecular biology studies.

### 4.3. Histomorphometry

Sections, 2 µm thick, of paraffin-embedded tissue were mounted on slides pretreated with 2% APES in acetone. Representative sections of 10 different segments of each artery were analyzed and histomorphometric results represent the average of 10 segments distributed over the entire artery. From each segment, six sections were obtained, one for hematoxylin–eosin staining for histology and histomorphometry, and the rest for immunohistochemistry. Sections with complete thrombosis of the vessel were discarded.

In hematoxylin–eosin-stained samples, the vessel lumen area, intimal hyperplasia area and media area were calculated with the Image Pro Plus quantitative image analysis system (Media Cibernetics, Rockville, MD, USA). The percentage of stenosis and the intima–media ratio (I/M) were calculated using standard equations: I/M = intimal area/media area; % stenosis = 100 × [intimal area/(lumen area + intimal area)] [59,60]. Results for each arterial sample represent the mean of all the sections analyzed.

### 4.4. Immunohistochemistry

Sections, 2 µm thick, of paraffin-embedded tissue were mounted on slides pretreated with 2% APES in acetone. Once deparaffinized and rehydrated in decreasing ethanol concentrations, sections were incubated for 30 min in 3% H_2_O_2_/methanol (1:1) to block endogenous peroxidase [61]. After washing in PBS, they were incubated for 1 h in 6% host serum for the secondary antibody, and 4% bovine serum albumin (BSA) in PBS. Then, they were incubated for 18 h at 4 °C with the primary antibodies (1:50 mouse anti-CD74 monoclonal antibody, (1:50, Santa Cruz Biotechnology, Dallas, TX, USA); polyclonal rat anti-CD43 antibody, (1:100, Beckton Dickinson, Franklin Lakes, NJ, USA) rabbit anti-NIK polyclonal antibody, (1:100, Cell Signaling, Danvers, MA, USA) in 1% serum and 4% BSA in PBS. Then, they were washed in PBS and incubated with biotin-conjugated secondary antibodies (1/200, Amersham, UK) in 4% BSA–PBS for 1 h at room temperature. Samples incubated with biotin-conjugated secondary antibody were treated with the ABC–HRPO complex (DAKO, Glostrup, Denmark) for 30 min at 37 °C and developed with DAB chromogen (Dako, Glostrup, Denmark): 3% H_2_O_2_ (130:1) for 5–10 min. Finally, sections were background stained with Carazzi hematoxylin (Bio-Optica, Milan, Italy), dehydrated and mounted in DPX medium (BDH, Poole, England). Negative controls were incubated without primary antibody. Immunohistochemistry was performed in sections, representing the median I/M value in the histomorphometry study for each sample.

### 4.5. Gene Expression Studies: Reverse Transcription and Real-Time Polymerase Chain Reaction

Total RNA was extracted by the Trizol Reagent method (Invitrogen, Carlsbad, CA, USA) and 1 µg RNA was reverse transcribed with High-Capacity cDNA Archive Kit (Applied Biosystems, Foster City, CA, USA). Quantitative PCR was performed in a 7500 Real Time PCR System with the ABI Prism 7500 System SDS software (v1.5.1) using predeveloped primers (GDF15: Mm00442228_m1, CD74: Mm00658576_m1, NIK: Mm00444166_m1 and MCP-1: Mm00441242_m1; all from Applied Biosystems) and RNA expression of different genes was corrected for GAPDH expression [24].

### 4.6. Statistical Analysis

The SPSS 11.0 statistical program was used. Data were expressed as mean ± standard deviation (mean ± SD) or median (interquartile range). Statistical significance at a level of *p* < 0.05 was established using Student’s *t* test for two groups of data and ANOVA for three or more groups. For the morphometric study, comparisons were made with non-parametric tests. The Mann–Whitney test was used for two-group comparisons, and the Kruskal–Wallis test used for three-group comparisons.

## Figures and Tables

**Table 1:** Histomorphometry of arterial injury. Data expressed as median (interquartile range).

	Sham Vehicle	Injury Vehicle	Sham Paricalcitol	Injury Paricalcitol	*p* Value (Injury Paricalcitol vs. Injury Vehicle)
N	5	5	5	4 *	
Intima/media ratio	0 (0, 0)	0.18 (0.11, 0.40)	0 (0, 0)	0.17 (0.07, 0.29)	0.41
% stenosis	0 (0, 0)	64.5 (55.1, 95.4)	0 (0, 0)	28.3 (6.6, 74.7)	0.11

* One sample could not be processed as it was thrombosed.

## Data Availability

The data used and/or analyzed during the current study are available from the corresponding author on reasonable request.
